# Cholecystomegaly: A Case Report and Review of the Literature

**DOI:** 10.1155/2020/8825167

**Published:** 2020-08-20

**Authors:** Mohammad Bagher Jahantab, Vahid Salehi, Saadat Mehrabi, Lotfolah Abedini, Mohammad Javad Yavari Barhaghtalab

**Affiliations:** General Surgery Department, Shahid Beheshti Hospital, Yasuj University of Medical Sciences, Yasuj, Iran

## Abstract

Chronic cholecystitis or symptomatic gallbladder is a prolonged mechanical or functional disorder of abnormal gallbladder emptying. Most of the patients have recurrent pain attacks (acute biliary colic), but when pain lasts more than 24 hours, it requires urgent surgical intervention (acute cholecystitis). The length of a fully distended gallbladder is about 7 to 10 cm. We report a case of a huge and severely inflamed gallbladder, as we have just found only a few previous case reports of the huge gallbladder in the literature. This case report and review may help to find a mechanism for the development of a giant gallbladder. The patient was a 36-year-old woman, who had been known to have a symptomatic gallstone for at least three years. The patient underwent laparotomy, and a giant 22 cm roundish severely inflamed and overdistended gallbladder with wall thickening and tight adhesion to adjacent organs was found under the right liver lobe. Femininity and diabetes seem to be risk factors for developing a huge gallbladder, and several hypotheses are encountered: (1) a long-lasting obstructed cystic duct or biliary tree, and accumulation of mucosal secretion from the gallbladder epithelium, (2) an obstructed hepatic/cystic duct junction with a stone acting like a check valve and bile trapping mechanism, and (3) gallbladder dysfunction and cholecystoparesis affecting through reduced cholecystokinin and celiac parasympathetic nerve disturbance in diabetes and diabetic autonomic neuropathy. Open cholecystectomy is the technique of choice in surgical excision of a huge gallbladder; however, laparoscopy could be performed by expert hands.

## 1. Background

Cholecystitis is the inflammation of the gallbladder. Symptoms include postprandial right upper quadrant or epigastric abdominal pain, and it is often called biliary colic. If biliary colic is left untreated, then most of the patients will develop chronic noninfectious inflammation of the gallbladder wall (chronic cholecystitis) [1]. When the pain lasts more than 24 hours without resolving, it can progress to a more severe form of cholecystitis (acute cholecystitis), so then requiring an urgent intervention [1, 2]. Contrary to the biliary colic, the pain of acute cholecystitis does not decrease [1].

In the etiology of cholecystitis, obstruction of the cystic duct by a gallstone or rarely an obstructive tumor is the initiating event that leads to distention, inflammation, edema, and mechanical dysfunction of the gallbladder. In this regard, the gallbladder wall becomes grossly thickened and reddish with subserosal hemorrhages and pericholecystic fluid. The mucosa may show hyperemia and patchy necrosis. Hypokinetic functioning of the gallbladder can also lead to a static state of gallbladder bile and is a precipitating factor for sludge and gallstone formation [1, 2]. Mucosal toxin lysolecithin (a product of lecithin), bile salts, platelet-activating factor, and an increase in prostaglandin synthesis may be associated with the inflammatory process in acute cholecystitis [1].

Cholecystectomy is the most common abdominal surgery in Western countries. Before the adventure of laparoscopic cholecystectomy by Philippe Mouret in 1987, an open technique was the standard procedure for cholecystectomy for more than 100 years (Carl Langenbuch made the first successful cholecystectomy in 1882). Now, the laparoscopic technique is the preferred method for the treatment of cholecystitis [2, 3].

The gallbladder is a pear-shaped sac that measures around 7 to 10 cm long and 4 cm wide, with an average capacity of 30 to 50 mL. When obstructed, the gallbladder can distend markedly and contain up to 300 mL of fluid [1, 4, 5]. We report a case with a huge and severely inflamed gallbladder, as we have just found only a few earlier case reports with a huge gallbladder in the literature [6–18]. This case report and review may help to find the mechanism for the development of a giant gallbladder or cholecystomegaly.

## 2. Case Presentation

The patient was a 36-year-old woman, a known case of symptomatic gallstone for at least three years. She was admitted to the emergency department at Yasuj Shahid Beheshti Hospital in April 2019, with a 2-day history of progressive increasing abdominal pain in the right upper quadrant with the radiation of pain to the epigastric area. The patient also had nausea, vomiting, and anorexia. She had 3 times previous admissions for the same problem last year, in which she did not give consent for operation and she did not use any medications.

On examination, she was hemodynamically stable and was not febrile. She had no jaundice, and the patient had abdominal tenderness in the right upper quadrant, and the gallbladder was palpable, with a positive Murphy's sign.

Laboratory tests showed a white blood cell (WBC) count of 9300 mm^3^, mild increase in total bilirubin, direct bilirubin, and aspartate aminotransferase (AST) (1.24 mg/dl, 0.68 mg/dl, and 36 u/l, retrospectively), a erythrocyte sedimentation rate (ESR) of 58.4 mm in one hour, and a C-reactive protein (CRP) of 58.4 mg/dL. Serum amylase, lipase, alanine transaminase (ALT), and alkaline phosphatase were all at normal levels. Urine analysis was normal.

**Figure 1:**
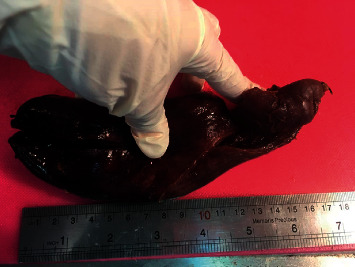
The huge gallbladder after fixation in formalin.

Abdominal sonography showed a dilated edematous gallbladder with a thickened wall more than 10 mm (the gallbladder size and dimensions were not included in the ultrasound report), with many small stones and sludge in which the largest one was about 25 mm and was indicative of acute cholecystitis. The common bile duct and hepatic ducts had normal caliber without any pathology. As the diagnosis was exact and there was no clinical suspicion for the complications of acute cholecystitis such as cholangitis or gallbladder perforation according to the ultrasound report, other diagnostic modalities such as computed tomography (CT) scan was not performed. The patient underwent open cholecystectomy through midline laparotomy on the day of admission. The findings of abdominal sonography were compatible with what we saw during laparotomy. A giant roundish severely inflamed and overdistended gallbladder with wall thickening and with tight adhesion to adjacent organs was found under the right liver lobe. Decompression of the gallbladder with needle aspiration was performed to decrease the size and make the cholecystectomy easier. After the decompression, a large impacted stone in the neck of the gallbladder was palpated. Cholecystectomy was performed successfully, and the exact size of the gallbladder was about 22 cm length, 6 cm width, and 1 cm thickness for the gallbladder wall.  Figure 1 shows the gross pathology of the specimen after fixation in formalin (the difference between the real postoperation and the gross pathology gallbladder size was due to the formalin shrinking effect).

The patient got well after the operation and was discharged in good condition on the fourth day of admission. Histopathological evaluation revealed chronic gangrenous cholecystitis, with no sign of malignant change. The patient was followed up in the next 4 weeks after the operation without any complications.

## 3. Discussion

Grosberg [6] reported the first published giant gallbladder in the literature. Bloom and Stachenfeld [7] discussed the diabetic cholecystomegaly observed in three cases for the first time. Cohen et al. [8] presented a case with acromegalic cholecystomegaly. Maeda et al. [9] resected a congenital form of the giant gallbladder. Tessier et al. [10] removed a huge gallbladder by laparoscopic surgery. Hsu et al. [11] reported the first and the only huge cancerous gallbladder in the literature. Panaro et al. [12] presented the largest gallbladder even reported in the world in a patient with Byler's disease (a group of diseases known as progressive familial intrahepatic cholestasis). The patient had chronic diarrhea and severe pruritus and was referred for liver transplantation. Taj [13] was issued in the Guinness Book of World Records as the first surgeon who operated the world's longest gallbladder through laparoscopic cholecystectomy at Capital Development Authority (CDA) hospital. Zong et al. [14] presented a huge congenital form of the gallbladder, which was marked as a giant celiac cyst before the operation. Kuznetsov et al. [15] reported the only huge gallbladder found in the literature without any evidence of acquired, congenital, and obstructive cause for the enlargement of the gallbladder. Edlin et al. [16] presented a patient with chronic cholecystitis who underwent cholecystostomy and then laparoscopic cholecystectomy. Kankaria et al. operated a huge gallbladder mucocele, belonged to Suman from Jaipur, India, through a laparoscopic approach, and the Guinness Book of World Records accepted it for the longest gallbladder in the world till now [17, 18].  Table 1 shows the huge gallbladders reported in the literature in the order of published year till now.

Our case study was comparable with the earlier studies according to size, procedure method, and pathology. All the patients were female except two diabetic cholecystomegaly patients who were male and in one whom sex identity was not mentioned. It shows that men develop less likely to have huge gallbladders except in diabetic patients. The patients' ages were from 17 to 95 years. According to the size, in Panaro's case report, the largest gallbladder even seen in the world till now was about 43 cm. However, the gallbladder size in our case was about 22 cm, and it was smaller than five previous reported studies [12–18]; it is large enough to be presented as a case report, as there are no defined borders proposed to distinguish between simply enlarged and giant gallbladders [15].

According to the procedure method used, Tessier et al. [10, 13, 16–18] performed laparoscopic cholecystectomy. In our study, classic open cholecystectomy was preferred as like as some other studies [6, 9, 11, 12, 14, 15]. In giant gall stones, it is much difficult to do laparoscopic cholecystectomy, so conversion from laparoscopic to open cholecystectomy is often performed because the giant stones are associated with more inflammation and thickening of the gallbladder wall and could make it much more difficult to hold the gallbladder with a grasper and expose Calot's triangle anatomy and retrieve such a large gallstone [19, 20]. Because of these whys and wherefores, open cholecystectomy is the ideal procedure in such cases [19]. However, this procedure is much difficult in these instances, but it is not an absolute indication for open cholecystectomy, and laparoscopy could be performed by an expert laparoscopic surgeon [20].

In the earlier case reports and our study, the dominant pathologies for giant gallbladders were as follows: chronic inflammation (cholecystitis), congenital, diabetes, visceromegaly due to acromegaly, inappropriate accumulation of mucus (mucocele), malignancy (adenocarcinoma), cholelithiasis, and cholestatic liver disease (Byler's disease). In our study, chronic inflammation (gangrenous cholecystitis) and an obstructive stone were the dominant pathologies. According to the existence of an obstructive stone in the gallbladder, our results were as same as Grosberg, Kankaria, and Tessier's studies [6, 10, 17, 18] in which a large impacted stone at the gallbladder neck was found. In the other studies, there was no obstructive etiology [8, 10–12, 14, 15]. In Bloom and Stachenfeld [7] and Zong's [14] case reports, as there was no inflammation and obstruction, it was considered to be a congenital etiology.

For the establishment of an overdistended huge gallbladder, several hypotheses could be identified: (1) The first process could be the hydrops of the gallbladder, in which an impacted stone without cholecystitis is present. Bile could not enter the gallbladder due to the long-lasting obstructed cystic duct or biliary tree, but the gallbladder epithelium will continue to secrete mucus and the gallbladder will become distended with clear-white mucinous material leading in the gallbladder wall edema, dilatation, inflammation, infection, and maybe perforation [1, 21, 22]. (2) The second hypothesis could be that the stones may move down and obstruct the hepatic/cystic duct junction acutely. One huge stone or multiple large stones act like a check valve allowing bile to enter but not exit (bile trapping) and impair drainage, and this leads to the slow resorption of the bile, distension, and enlargement of the gallbladder. When the disease progresses, the giant gallbladder became filled with bile, which leads to swelling and inflammation and targets the contractile ability of the gallbladder and can cause the gallbladder to grow large [14, 22]. (3) Another hypothesis is associated with physiological gallbladder contraction and relaxation which are regulated by both the endocrine and autonomic nervous system. Cholecystokinin (CCK) stimulates the gallbladder to contract and release stored bile into the intestine, and the parasympathetic nerve evokes gallbladder evacuating [23]. It has been shown that postprandial plasma cholecystokinin secretion and maximal gallbladder emptying after feeding were much reduced during hyperglycemia [24]. In patients with diabetic autonomic neuropathy, cholinergic action would be diminished by disturbance of the celiac parasympathetic nerve, resulting in gallbladder dysfunction and cholecystoparesis [23], so we could hypothesize that cholecystomegaly could occur in conjunction with diabetes and diabetic autonomic neuropathy through gallbladder dysfunction and cholecystoparesis.

The strength of this study is its comprehensive literature review, and its limitation is that we just check English literature for this review. Femininity and diabetes seem to be risk factors for developing a huge gallbladder. Conditions in which gallbladder increases in size are inflammation, acromegaly, diabetes, congenital, mucocele, malignancy, cholelithiasis, and intrahepatic cholestasis. Large or giant gallbladders need special surgical intervention. Open cholecystectomy is the technique of choice in surgical excision of a huge gallbladder, but laparoscopy could be performed, too by expert hands. The primary take away lesson of this report is to take enough time for our patients and give enough information to convince them to operate in a proper time to prevent a difficult, time-consuming, and high-risk operation instead of an easy, rapid, and low-risk surgery.

## Figures and Tables

**Table 1:** The huge gallbladders reported in the literature in the order of published year till now.

	Year	Country	Author	Age (years)	Sex	Gallbladder size	Kind of operation	Pathology
1	1962	USA	Grosberg [6]	95	Female	14 cm	Open cholecystectomy	Acute gangrenous cholecystitis
2	1969	USA	Bloom and Stachenfeld [7]	61/66/70	Female/male/male	3 to 9 times normal size	Nonoperative management	Three cases of diabetic cholecystomegaly
3	1974	USA	Cohen et al. [8]	65	Female	18 cm	Nonoperative management	Visceromegaly as a feature of acromegaly
4	1979	Japan	Maeda et al. [9]	36	Female	18 cm	Open cholecystectomy	Neither inflammatory nor obstructive marked as a congenital giant gallbladder
5	2005	USA	Tessier et al. [10]	78	Female	17 cm	Laparoscopic cholecystectomy after needle decompression	Stone impaction and dilatation in the neck of the gallbladder, acute and chronic cholecystitis
6	2011	Taiwan	Hsu et al. [11]	87	Female	16.4 cm	Emergency laparotomy with extended cholecystectomy	Acute suppurative cholecystitis with a fungating tumor showing poorly differentiated adenocarcinoma
7	2012	France	Panaro et al. [12]	17	Not available (N/A)	43 cm	Open cholecystectomy at the time of liver transplantation	Byler's (intrahepatic cholestasis), no pathologic change in the gallbladder
8	2012	Pakistan	Taj [13]	70	Female	25.5 cm	Laparoscopic cholecystectomy	N/A
9	2013	China	Zong et al. [14]	55	Female	30 cm	Open cholecystectomy	No obstructive cause, so congenital giant gallbladder considered
10	2014	Russia	Kuznetsov et al. [15]	77	Female	24 cm	Open cholecystectomy	No acquired, congenital and obstructive cause
11	2015	United Kingdom	Edlin et al. [16]	88	Female	12 cm	Cholecystostomy and then laparoscopic cholecystectomy 6 weeks later	Chronic cholecystitis
12	2017	India	Kankaria et al. [17, 18]	46	Female	30 cm	Laparoscopic cholecystectomy	Chronic cholecystitis, cholelithiasis, gallbladder mucocele with a large impacted stone
13	2019	Iran	Jahantab (current report)	36	Female	22 cm	Open cholecystectomy	Chronic gangrenous cholecystitis

## Data Availability

The data used to support the findings of this study are available from the corresponding author upon request.

## Abbreviations

WBC: White blood cell
CT: Computed tomography
AST: Aspartate aminotransferase
CRP: C-reactive protein
ALT: Alanine transaminase
CDA: Capital Development Authority hospital
N/A: Not available
CCK: Cholecystokinin.
